# A 25-year Landsat-derived environmental observation dataset for Narayanganj, Bangladesh, 2001–2025

**DOI:** 10.1016/j.dib.2026.113392

**Published:** 2026-10-30

**Authors:** Joynal Abadin, ASM Shihavuddin, Aminul Islam

**Affiliations:** a Department of Electrical and Electronic Engineering, Green University of Bangladesh, Purbachal American City, Kanchan, Rupganj, Narayanganj 1461, Dhaka, Bangladesh; b Department of Electrical and Electronic Engineering, University of Liberal Arts Bangladesh, 688 Beribadh Road, Dhaka, Mohammadpur 1207, Bangladesh; c Department of Civil and Mechanical Engineering, Technical University of Denmark, Koppels Allé, Building 404, DK-2800 Kgs. Lyngby, Denmark

**Keywords:** Surface reflectance, Google Earth Engine, Land surface temperature, Urban environment, Remote sensing

## Abstract

A point-based environmental observation dataset for Narayanganj, Bangladesh, is introduced in this work, based on Landsat Collection 2 Tier 1 Level-2 imagery available on Google Earth Engine. A total of 3591,676 quality-masked pixel observations at 5000 spatial sample points were extracted from 1415 Landsat 5, 7, 8, and 9 images between January 2001 and December 2025. Each year of data is contained within a CSV file providing surface reflectance bands, thermal measurements, land surface temperature, water occurrence, QA_PIXEL, QA_RADSAT, ST_QA, cloud cover, solar geometry, WRS path/row, product names, coordinates, observation date, and mission information. A supporting workflow script has been developed to process data from various missions by applying mission-specific preprocessing, radiometric scaling, QA_PIXEL and optical QA_RADSAT masking, band-name harmonisation to a common variable schema, and annual export steps. This dataset can be used to support reproducible monitoring of the urban environment, assess land cover and land surface temperature, philtre water-covered areas, and develop machine-learning applications in Narayanganj District, which is undergoing accelerated urbanisation. The same pipeline can also be adapted to other study areas by modifying the relevant spatial and temporal parameters

Specifications Table

**Table:** 

Subject	Earth & Environmental Sciences
Specific subject area	Multi-decadal satellite-derived environmental observations for urban and peri‑urban monitoring in Narayanganj, Bangladesh
Type of data	Processed tabular data (CSV); source code (JavaScript and Python); figures (PNG); assessment report (TXT)
Data collection	Landsat Collection 2 Tier 1 Level-2 images intersecting the Narayanganj region of interest were filtered by mission-specific operational periods, scaled to surface reflectance and thermal units, masked using QA_PIXEL and optical QA_RADSAT, sampled at 5000 points, and exported annually from Google Earth Engine. QA_PIXEL, QA_RADSAT, and ST_QA are retained in the CSV files so that users can apply additional philtres.
Data source location	Narayanganj District, Bangladesh; approximate coordinate range of the exported sampled pixel centres: 90.4077–90.8031°E and 23.5739–23.9482°N
Data accessibility	Repository name: Zenodo Data identification number: https://doi.org/10.5281/zenodo.22216717 Direct URL to data: https://zenodo.org/records/22216717 Instructions for accessing these data: The dataset is freely and publicly accessible at the above URL; no registration is required.
Related research article	None

## 1 Value of the Data

• The dataset provides a 25-year Landsat record for Narayanganj in a common variable schema, enabling reproducible analysis of long-term urban environmental change. Harmonisation here refers to band names, scale factors, and table structure, not to inter-sensor spectral calibration.
• The annual CSV structure and 5000 repeated sample points support time-series modelling, trend analysis, and machine-learning workflows.
• The inclusion of optical bands, land surface temperature, cloud cover, QA_PIXEL, QA_RADSAT, ST_QA, water occurrence, product identifiers, and solar geometry allows users to apply alternative filtering, validation, and feature-engineering strategies.
• The dataset is useful for researchers studying urban heat [1], surface-water dynamics [2], land-cover change, climate exposure, and remote-sensing methods [3,4] in rapidly urbanising South Asian contexts.
• The Google Earth Engine extraction script and assessment outputs provide a transparent basis for reproducing, auditing, or extending the dataset to other years or study areas.

## 2 Background

Long-term Earth observation data can provide crucial insight into environmental, urban, and surface thermal trends. The publicly available Landsat satellite archive, combined with cloud-based data processing and storage platforms such as Google Earth Engine, provides an avenue to produce consistent observations of the Earth's surface with standardised preprocessing and quality control. In contrast, environmental datasets for rapidly urbanising areas such as Bangladesh are fragmented across individual scenes and require extensive preprocessing before use. This dataset was produced to provide an annualised view of environmental observations for the case study area of Narayanganj District, Bangladesh, spanning from 2001 to 2025. The data-generation pipeline was designed to process imagery from Landsat 5, 7, 8, and 9 Collection 2 Tier 1 Level-2 products through individual mission-specific radiometric scaling, QA_PIXEL and optical QA_RADSAT masking, band-name harmonisation to a common variable schema, fixed-point sampling, and annual CSV export procedures. The generated CSV files contain surface reflectance values, land surface temperature, long-term water occurrence, image metadata, QA_PIXEL, QA_RADSAT, ST_QA, and acquisition parameters for each year using a uniform set of processing steps. The accompanying Google Earth Engine script and this article describe the data-generation procedures in detail and facilitate replication of the pipeline by other researchers.

## 3 Data Description

The dataset was developed to present a consistent historical environmental dataset for Narayanganj District over a 25-year period by using the same sampling locations each year. A complete 25-year dataset consists of 25 individual year-level CSV data files, one for each year from 2001 to 2025 inclusive. Over the years covered, the assessment report identifies 3591,676 observations, 1415 unique Landsat images, and 5000 unique sample locations.

Temporal coverage is complete without any missing years from January 2001 until December 2025. Table 1 shows the yearly count of observations and the number of contributing Landsat images for each year.

**Table 1:** Annual dataset summary from the assessment report.

Year	2001	2002	2003	2004	2005	2006	2007	2008	2009
Observations	84,424	77,872	69,221	114,804	126,598	147,033	101,013	139,745	140,671
Images	38	29	29	54	51	58	45	66	58
Year	2010	2011	2012	2013	2014	2015	2016	2017	2018
Observations	108,415	144,946	76,415	109,276	164,599	193,599	137,540	169,697	196,146
Images	48	53	33	45	67	68	61	65	73
Year	2019	2020	2021	2022	2023	2024	2025		
Observations	162,672	152,561	168,129	223,640	205,357	177,253	200,050		
Images	65	56	67	80	68	64	74		

The accompanying data package comprises 25 annual CSV files named Narayanganj_EnvDataset_2001.csv through Narayanganj_EnvDataset_2025.csv, the Google Earth Engine JavaScript extraction script (gee_extraction_corrected.js), the Python assessment script (narayanganj_dataset_assessment.py), the plain-text assessment report (Narayanganj_Dataset_Assessment_Report.txt), and two PNG summary figures (Temporal_Scene_Distribution_and_Cloud_Cover.png and Landsat_Mission_Data_Composition.png) [5].

Yearly counts of observations varied between 69,221 in 2003 and 223,640 in 2022. The variation between the years shown in Table 1 is explained by the number of imagery records acquired, the overlapping of different missions, cloud cover, quality flags, and finally, the number of quality-masked observations retained after preprocessing. Calendar-year coverage is complete for 2001–2025, but completeness is not equal at every sampling location. Across the 5000 Point_IDs, the number of retained observations ranges from 408 to 970 (25th percentile 470; median 718; 75th percentile 911; mean 718.3). The statement that the dataset contains no missing values refers only to the absence of null cells in retained rows, not to equal temporal coverage at every Point_ID. Users conducting time-series or trend analyses should account for this per-location variability. Since the fields in the CSV are the same for each year, the CSV files for different years can be concatenated or post-processed using the same script/philtre. The Water_Occurrence_Pct field represents the percentage of valid JRC Global Surface Water observations from 1984 to 2021 in which the sampled pixel was classified as water; values near 0 indicate rarely or never detected water, whereas higher values indicate more persistent water occurrence. The fields that were exported are documented in Table 2, including description, unit, data type, source, expected range, and interpretation of the QA fields. The fields comprise identifiers, time variables, mission and processing metadata, solar and spatial metadata, optical reflectance, thermal and water variables, and pixel- and scene-level quality fields (QA_PIXEL, QA_RADSAT, ST_QA, and CLOUD_COVER).

**Table 2:** Data dictionary for fields in the annual CSV files.

Field Name	Description	Data Type	Units	Source	Expected Range	QA Interpretation & Guidance
**Observation_ID**	Unique observation key	String	—	GEE script	—	—
**Point_ID**	Fixed sample location	Integer	—	GEE script	0–4999	—
**Image_ID**	Scene identifier	String	—	Landsat metadata	—	—
**Landsat_Product_ID**	USGS Level-2 product ID	String	—	Landsat metadata	—	—
**Observation_Date**	Acquisition date	Date	YYYY-MM-DD	Landsat metadata	2001–01–05 to 2025–12–25	—
**Acquisition_Timestamp**	Acquisition time	Integer	ms	Landsat metadata	—	—
**Landsat_Mission**	Satellite platform	String	—	Landsat metadata	LANDSAT_5, 7, 8 or 9	—
**Processing_Level**	USGS processing level	String	—	Landsat metadata	L2SP	—
**Collection_Category**	USGS collection category	String	—	Landsat metadata	T1	—
**WRS_Path**	WRS-2 path	Integer	—	Landsat metadata	137	—
**WRS_Row**	WRS-2 row	Integer	—	Landsat metadata	43 or 44	—
**Sun_Azimuth**	Solar azimuth	Float	°	Landsat metadata	45.0–150.0	—
**Sun_Elevation**	Solar elevation	Float	°	Landsat metadata	30.0–65.0	—
**Longitude**	Pixel-centre longitude	Float	°E	GEE script	90.41–90.80	—
**Latitude**	Pixel-centre latitude	Float	°N	GEE script	23.57–23.95	—
**Blue, Green, Red, Near_Infrared, SWIR1, SWIR2**	Surface reflectance	Float	—	Landsat L2 SR	−0.05 to 1.05	Exclude values outside this range.
**Thermal_Band_Raw**	Unscaled thermal DN	Integer	DN	Landsat L2 ST	293–57,222	Used to derive LST.
**Land_Surface_Temperature**	Land surface temperature	Float	°C	Derived	0–60	Exclude 〈 0 or 〉 60 °C.
**Water_Occurrence_Pct**	JRC water occurrence	Integer	%	JRC GSW v1.4 [2]	0–100	0 = never water; 100 = permanent.
**CLOUD_COVER**	Scene cloud cover	Float	%	Landsat metadata	0–90	Scene-level; not pixel cloud fraction.
**QA_PIXEL**	Pixel quality bitmask	Integer	Bitmask	Landsat L2 QA	—	Fill, cloud, shadow (cirrus on L8/9) masked at extraction.
**QA_RADSAT**	Saturation bitmask	Integer	Bitmask	Landsat L2 QA	0	Optical saturation masked at extraction. Exclude ≠ 0 if accuracy is critical.
**ST_QA**	LST uncertainty	Integer	DN (×0.01 K)	Landsat L2 QA	—	Uncertainty (K) = DN × 0.01. For precision work, retain < 2 K.

Fig. 1 summarises the annual availability of Landsat scenes and their associated mean cloud cover characteristics across the 2001–2025 study period. The temporal variation reflects differences in satellite mission availability, image acquisition frequency, and quality-filtered observations after QA-based masking. The transition from Landsat 5 and 7 to Landsat 8 and 9 is clearly visible, while the relatively stable annual scene availability demonstrates the consistency of the generated dataset for long-term environmental analysis and reflects the continuity of the freely available global Landsat archive [3,4,6,7]. Surface reflectance observations from Landsat 8 and 9 are also expected to be highly consistent because of the close agreement between the two platforms demonstrated during underfly validation [8]. The accompanying cloud-cover statistics further assist users in selecting appropriate observations for quality-sensitive applications.

**Fig. 1:**
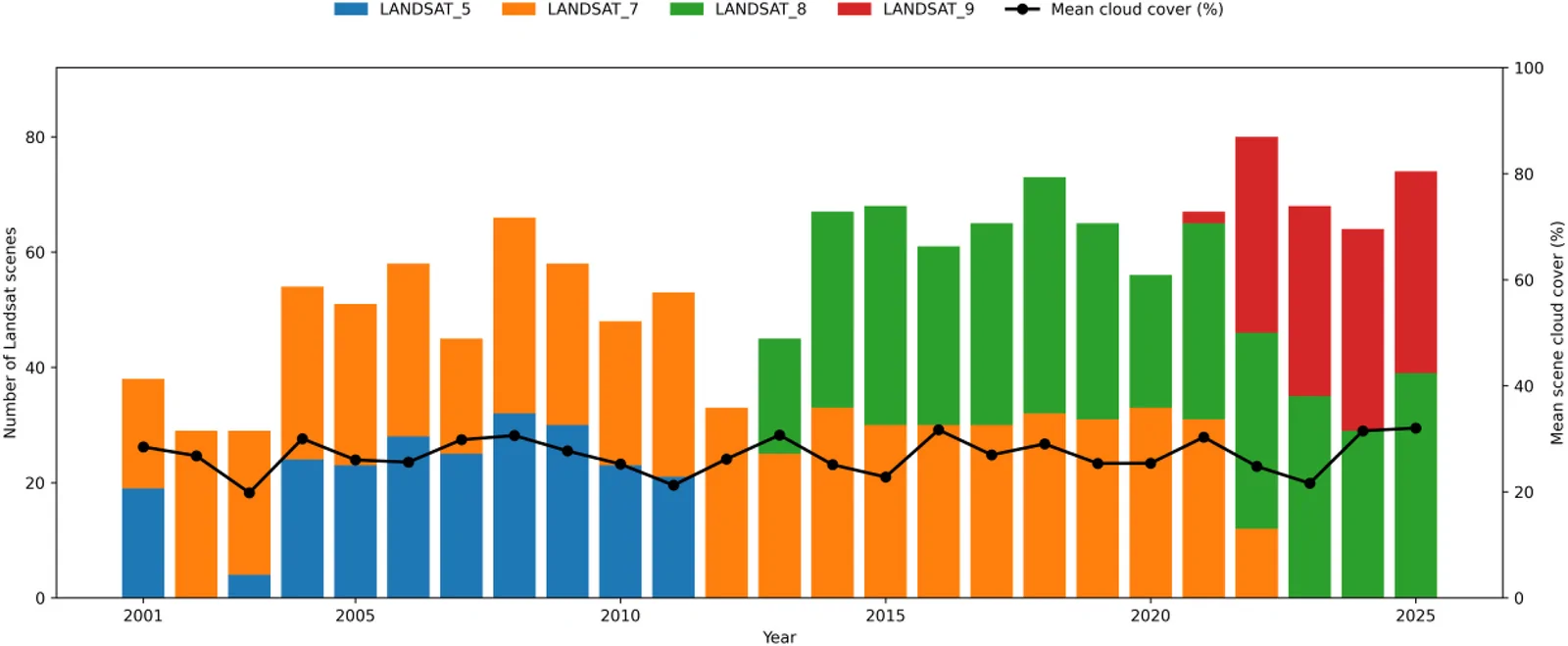
Temporal scene distribution and cloud-cover characteristics across the Landsat image archive used for the dataset.

Mission composition is another important consideration for reuse because the sensor mix changes across the 25-year archive. Fig. 2 therefore follows the temporal summary and identifies the proportional contribution of each Landsat mission to the complete dataset. In the generated Narayanganj dataset, Landsat 7 contributes the largest proportion of observations, followed by Landsat 8, Landsat 5, and Landsat 9; this ranking is influenced by Landsat 7′s long operational period and extensive archive [3]. This distribution reflects the historical evolution of the Landsat programme and provides users with a clear understanding of the temporal composition of the dataset.

**Fig. 2:**
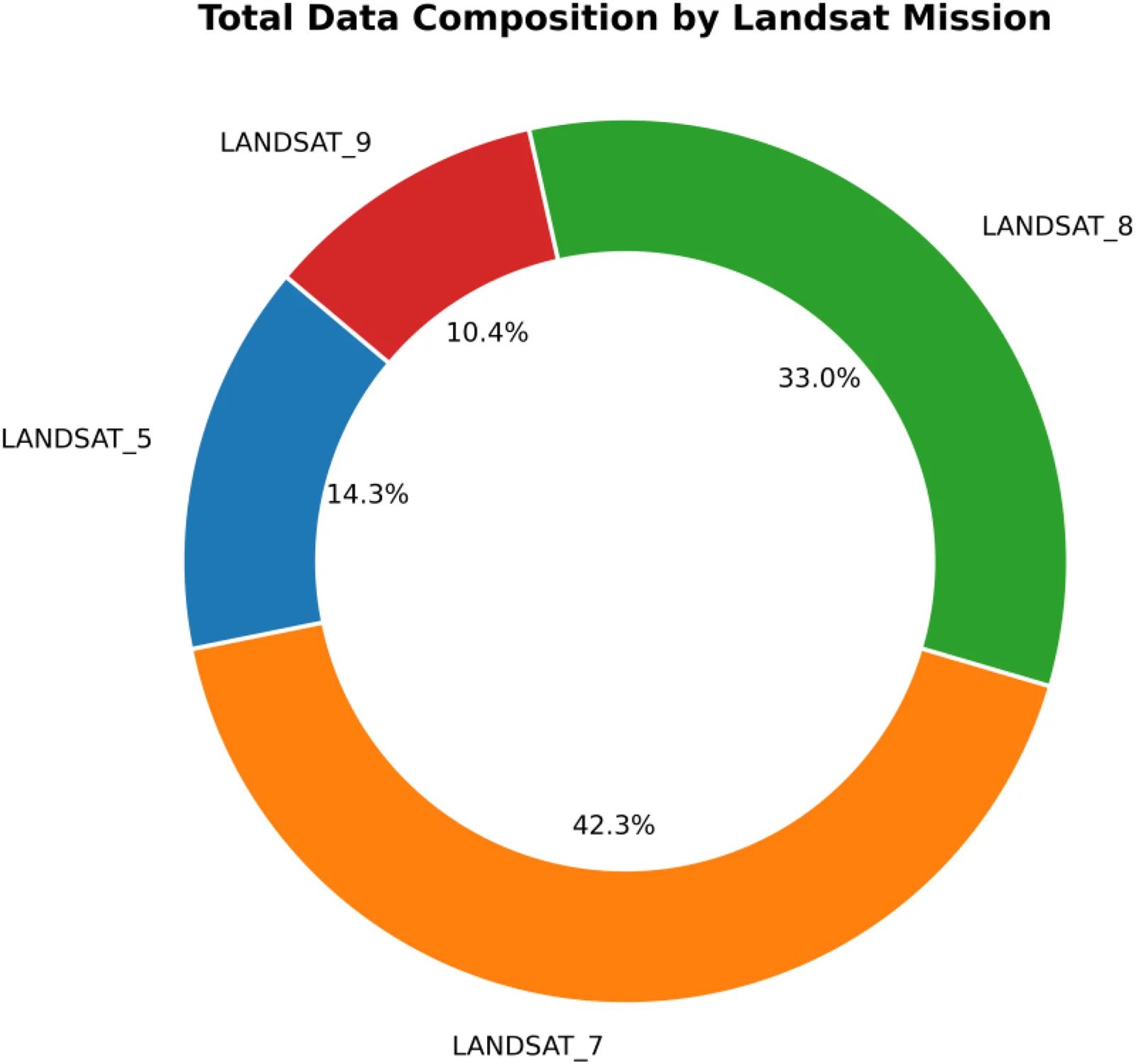
Composition of the complete dataset by Landsat mission.

## 4 Experimental Design, Materials and Methods

Fig. 3 presents the data-generation workflow used to transform Landsat Collection 2 Level-2 imagery into annual point-observation tables. The workflow highlights the sequence of defining the region of interest, generating a fixed set of random sample points, preparing Landsat imagery, extracting pixel values and metadata, and exporting annual CSV datasets.

**Fig. 3:**
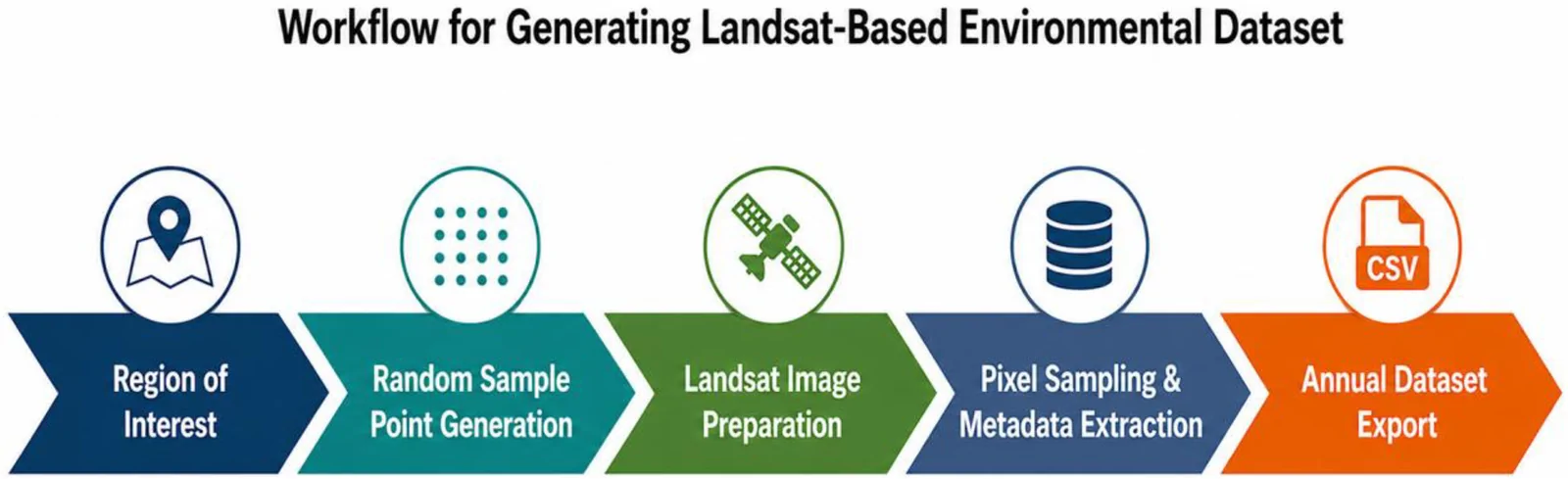
Workflow used to construct the Narayanganj Landsat environmental observation dataset.

### 4.1 Study area and sampling design

The described dataset was obtained for the Narayanganj District, Bangladesh. Within the administrative boundary of the region, five thousand points were randomly sampled in Google Earth Engine using the ee.FeatureCollection.randomPoints() function with a random seed of 42. The same set of points was used across all years (2001–2025). Five thousand points were chosen as a reproducible sample that is large enough for district-scale time-series, trend, and machine-learning use, while remaining computationally tractable for 25 years of multi-mission Google Earth Engine exports. The points were drawn as a simple random sample (ee.FeatureCollection.randomPoints(), seed 42) inside the FAO GAUL Narayanganj administrative boundary so that urban, peri‑urban, agricultural, and riverine surfaces present in the district could be included without imposing an external land-cover classification on the dataset. Independent land-cover maps can be joined to the exported Longitude and Latitude fields if class-specific subsets are required. The static JRC water-occurrence attribute shows that the sample is not restricted to one surface type: 71.2% of observations occur at locations never detected as water, 20.6% at rarely inundated locations (1–24% occurrence), and the remainder at seasonally to mostly water-covered locations [2]. By doing so, it becomes possible to approximate trends for each sample obtained from different datasets (Landsat satellites). Each fixed point was assigned a unique Point_ID when the sample collection was created, and each exported observation included that Point_ID together with the sampled pixel-centre longitude and latitude. The figure below demonstrates the extent of the region used as a context for sampling and extracting data (Fig. 4).

**Fig. 4:**
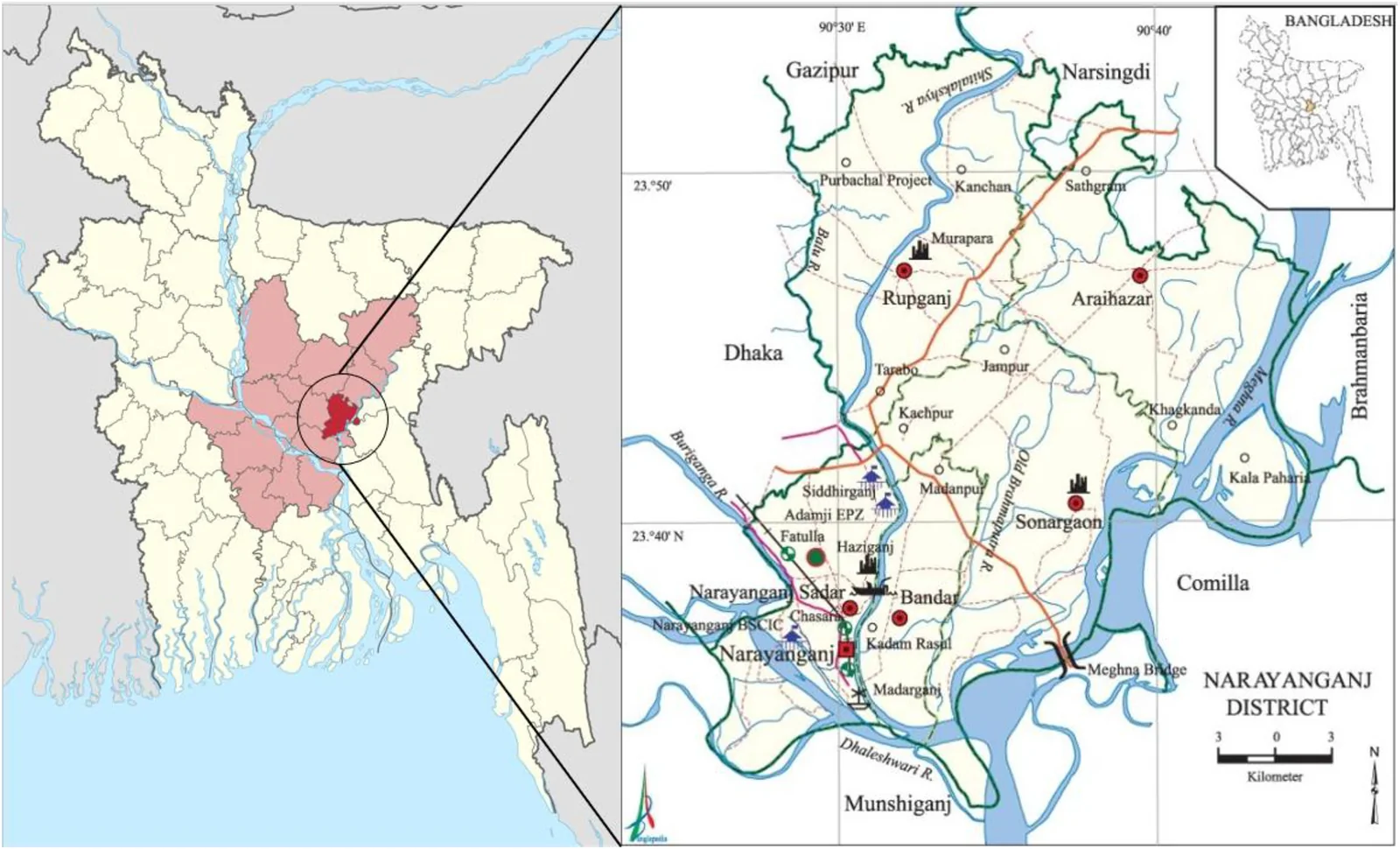
Study area map for Narayanganj, Bangladesh, showing the spatial context used for sampling and extraction.

### 4.2 Satellite data sources

The entire process started by defining the region of interest using the FAO Global Administrative Unit Layers (GAUL) dataset to obtain the Narayanganj District administrative boundary. Landsat Collection 2 Tier 1 Level-2 surface reflectance and surface temperature products were then accessed for Landsat 5 (LANDSAT/LT05/C02/T1_L2), Landsat 7 (LANDSAT/LE07/C02/T1_L2), Landsat 8 (LANDSAT/LC08/C02/T1_L2), and Landsat 9 (LANDSAT/LC09/C02/T1_L2) over the period January 2001–December 2025, considering their respective mission periods. The optical and surface temperature bands were converted to physical units using the respective USGS scale factors, followed by removal of poor-quality pixels using the QA_PIXEL band and of optically saturated pixels using QA_RADSAT. Mission-specific preprocessing brought all data into a consistent band nomenclature, after which pixel values were sampled at the fixed points and exported as one CSV file for each year. The entire workflow was implemented in the Google Earth Engine Code Editor using the JavaScript API, and the code is available in the Zenodo repository.

### 4.3 Preprocessing and harmonisation

Landsat Collection 2 scale factors were applied to optical and thermal bands using the per-pixel algebraic operations provided by the Google Earth Engine API [9]. The following expressions were used for optical surface reflectance: (0.0000275 × DN) − 0.2 and for surface temperature in Kelvin: (0.00341802 × DN) + 149.0; 273.15 was then subtracted to obtain degrees Celsius [10]. Landsat 5 and 7 surface reflectance bands (SR_B1–SR_B5 and SR_B7) were renamed to Blue, Green, Red, Near_Infrared, SWIR1, and SWIR2, while ST_B6 was retained as the unscaled Thermal_Band_Raw band and used to derive Land_Surface_Temperature. Landsat 8 and 9 were processed by renaming SR_B2–SR_B7 to the same six optical bands, while ST_B10 was retained and processed in the same way. Finally, all datasets were standardised to a common variable schema comprising optical reflectance bands, thermal measurements, land surface temperature, acquisition metadata, quality fields, and spatial characteristics across the 25-year Landsat archive. This step harmonises names, scaling, and table structure only; no spectral-response adjustment or radiometric cross-calibration among Landsat 5, 7, 8, and 9 was applied.

### 4.4 Quality masking

Quality assurance at extraction used the Collection 2 QA_PIXEL and QA_RADSAT bands [10,11]. Mission-specific QA_PIXEL masks were applied for Landsat 5/7 and Landsat 8/9. Fill, dilated cloud, cloud, and cloud-shadow pixels were excluded for all missions; cirrus-contaminated pixels were additionally excluded for Landsat 8 and Landsat 9. Optical QA_RADSAT bits were also masked (Landsat 5/7 bits 0–4 and 6; Landsat 8/9 bits 1–6) so that radiometrically saturated optical pixels were not exported. The thermal saturation bit was not used as an extraction mask. ST_QA was not used to drop observations; it is exported so that users can philtre on LST uncertainty, where uncertainty (K) = ST_QA (DN) × 0.01 [10]. QA_PIXEL, QA_RADSAT, and ST_QA are all written to the annual CSV files. Remaining out-of-range reflectance and physically implausible LST values were retained deliberately, so that users can apply application-specific philtres rather than a single hard purge at extraction. Recommended criteria are: (i) for reflectance analyses, exclude any optical band 〈 −0.05 or 〉 1.05 [10]; (ii) for LST analyses in Narayanganj, exclude Land_Surface_Temperature 〈 0 °C or 〉 60 °C; (iii) where radiometric accuracy is critical, exclude QA_RADSAT ≠ 0; and (iv) for LST precision work, retain observations with ST_QA-derived uncertainty < 2 K. The 0–60 °C LST limits are physical-plausibility thresholds for this study area. They are distinct from the IQR outlier fences in the assessment report, which are statistical diagnostics only and are not used as exclusion rules.

### 4.5 Annual export structure

After preprocessing, quality masking, and band-name / schema harmonisation, Landsat imagery from all missions was combined into a single image collection to extract pixel values for the fixed sample locations with their associated acquisition metadata, including Landsat mission, Landsat product, processing level, collection category, WRS path and row, observation date, acquisition time, cloud cover, QA_PIXEL, QA_RADSAT, ST_QA, solar azimuth and elevation, optical bands, thermal measurements, land surface temperature, water occurrence, and geographic coordinates. One CSV file per year was exported from Google Earth Engine for 2001–2025. In total, the 25 CSV files contain 3591,676 quality-masked pixel observations from 1415 Landsat scenes at 5000 fixed points within the study area.

### 4.6 Technical validation

A comprehensive dataset assessment was performed after generation of the annual CSV files to verify integrity, consistency, and reuse-related quality. The assessment confirmed that all 25 expected annual datasets (2001–2025) were generated without calendar-year gaps and that the complete dataset contains 3591,676 observations from 1415 unique Landsat scenes at 5000 fixed sampling locations. No missing cells, duplicate Observation_IDs, or fully duplicated rows were detected among retained records. The absence of missing cells refers only to null values in exported rows; it does not imply equal temporal completeness at every Point_ID. Retained observation counts per Point_ID ranged from 408 to 970 (25th percentile 470; median 718; 75th percentile 911; mean 718.3; standard deviation 197.1). This unevenness reflects mission availability, overlapping WRS tiles, scene cloudiness, QA_PIXEL and optical QA_RADSAT masking, and Landsat 7 Scan Line Corrector (SLC)-off gaps after May 2003. Fill pixels were excluded, so SLC-off does not insert null reflectance values, but it reduces the number of valid samples at some locations.

Image-level CLOUD_COVER ranged from 0.0% to 90.0% (mean 27.14%; median 17.4%). Of the 1415 scenes, 581 (41.1%) had scene cloud cover of 0–10%. Residual cloud or cloud-shadow contamination can remain because CLOUD_COVER is scene-level metadata and QA_PIXEL masking is imperfect [11]. Optical QA_RADSAT masking removed saturated optical pixels at extraction; only 6 retained observations (0.00%) have QA_RADSAT ≠ 0, consistent with non-optical or thermal flags that were not used as an extraction mask. ST_QA was evaluated but not applied as an exclusion mask. Converted LST uncertainty (ST_QA × 0.01) has a mean of 3.289 K and a median of 2.730 K; 10.5% of observations have uncertainty < 2 K and 44.0% have uncertainty > 3 K.

A small fraction of scaled surface-reflectance values fall outside 0–1, including 1004 observations with at least one optical band < −0.05. These values can result from the Collection 2 scale offset (DN × 0.0000275 − 0.2), dark water or residual shadow, or highly reflective surfaces, and are not necessarily processing errors [10]. Land surface temperature ranges from −123.15 °C to 71.44 °C. The lower extreme corresponds to very low thermal DNs (Thermal_Band_Raw minimum = 293) that passed pixel-quality masking and convert to physically implausible temperatures; 6669 observations (0.19%) are < 0 °C and 44 observations are > 60 °C. These records were retained so that users can apply the philtres given in Quality masking. The 0–60 °C LST limits are physical-plausibility thresholds for Narayanganj and must not be confused with the IQR fences in the assessment report.

## Limitations

The dataset is generated from observations collected at 5000 specific points, rather than wall-to-wall raster coverage, and the sample was not stratified by land-cover class. Users requiring class-specific analyses should overlay an independent land-cover map on the exported coordinates. Although Collection 2 Level-2 pixels were masked using mission-specific QA_PIXEL flags and optical QA_RADSAT bits, residual cloud or cloud-shadow contamination can remain [11]. Moreover, the CLOUD_COVER field is scene-level metadata and should not be confused with the cloud condition of the sampled pixel. Due to the failure of Landsat 7′s SLC in May 2003, some scenes have a reduced valid-pixel footprint, which lowers the number of retained observations at some Point_IDs; fill pixels themselves were excluded and do not appear as nulls. Band names, scale factors, and the CSV schema were standardised across Landsat 5, 7, 8, and 9; this is schema harmonisation only. No spectral-response or radiometric cross-calibration was applied, so residual inter-sensor differences can remain. A small number of scaled reflectance values fall below 0 or above 1 because of the Collection 2 offset (DN × 0.0000275 − 0.2), dark water or residual shadow, or highly reflective surfaces. Extreme LST values (−123.15 °C to 71.44 °C) are associated with residual invalid or near-fill thermal DNs that passed pixel-quality masking (Thermal_Band_Raw minimum = 293). These reflectance and LST extremes were retained so that users can apply application-specific philtres. Recommended thresholds are: exclude any optical band 〈 −0.05 or 〉 1.05; exclude Land_Surface_Temperature 〈 0 °C or 〉 60 °C for Narayanganj; exclude QA_RADSAT ≠ 0 where radiometric accuracy is critical; and, for LST precision work, retain observations with ST_QA-derived uncertainty < 2 K. The 0–60 °C limits are physical-plausibility thresholds for this study area and are distinct from the statistical IQR fences in the assessment report.

## Ethics Statement

The authors confirm that the present manuscript complies with the ethical requirements for publication in Data in Brief. This research did not involve human participants, human subject data, or animal experiments. The authors also confirm that no data were obtained from social media platforms. The dataset was created using publicly available remote-sensing satellite imagery. In addition, the dataset does not contain any personally identifiable information.

## Credit Author Statement

**Joynal Abadin:** Conceptualization, Methodology, Software, Data curation, Formal analysis, Validation, Visualization, Writing – original draft. **ASM Shihavuddin:** Methodology, Supervision, Project administration, Validation, Writing – review & editing. **Aminul Islam:** Validation, Formal analysis, Writing – review & editing.

## Declaration of Generative AI and AI-Assisted Technologies in the Manuscript Preparation Process

During the preparation of this work, the authors used Prism and Claude (Anthropic) for language editing and structural refinement. No AI tools were used for data generation, analysis, or figure processing. The authors reviewed and edited the output as needed and take full responsibility for the content of the published article.
